# The interplay of ferroptosis and oxidative stress in pulmonary fibrosis: from mechanisms to treatment

**DOI:** 10.3389/fcell.2026.1709805

**Published:** 2026-03-09

**Authors:** Peishuo Yan, Junjie Wang, Zhen Lei, Hongbo Chang, Weili Shi, Shangzeng Wang

**Affiliations:** 1 Henan Province Hospital of Chinese Medicine (The Second Affiliated Hospital of Henan University of Chinese Medicine), Zhengzhou, China; 2 The Second Clinical Medical College, Henan University of Chinese Medicine, Zhengzhou, China

**Keywords:** ferroptosis, lipid peroxidation, lung fibrosis, metabolic disorder, pathogenesis

## Abstract

Pulmonary fibrosis (PF) is a progressive and devastating interstitial lung disease characterized by the dynamic imbalance of multiple cell types and signaling pathways. In recent years, ferroptosis, an iron-dependent form of programmed cell death driven by lipid peroxidation, has been recognized as playing a significant role in the progression of pulmonary fibrosis due to its central role in oxidative stress, metabolic dysfunction, and disruption of barrier integrity. Existing studies have elucidated the core signaling pathways, key molecules, and potential roles of ferroptosis in PF progression, highlighting the synergistic pathogenic effects of iron homeostasis disruption and lipid peroxidation. Despite its established role in fibrosis, a comprehensive analysis of the cell-type-specific mechanisms of ferroptosis within pulmonary cell populations remains lacking. Furthermore, several small-molecule inhibitors targeting ferroptosis have demonstrated promising anti-fibrotic effects in animal models, yet their tissue-specificity, safety profiles, and clinical feasibility warrant further investigation. This review systematically summarizes the cell-type-specific roles of ferroptosis in PF, delineates the key molecular mechanisms and potential druggable targets involved, and underscores the potential of ferroptosis as a critical regulatory node at the intersection of metabolism and cell fate. By bridging the understanding of metabolic regulation and cell death processes, ferroptosis holds promise for providing novel mechanistic insights and informing precise therapeutic strategies for pulmonary fibrosis.

## 1 Introduction

Pulmonary fibrosis refers to a heterogeneous group of interstitial lung diseases characterized by persistent scarring and structural remodeling of lung tissue. The pathological core involves repeated micro-injury to alveolar epithelial cells, abnormal activation of fibroblasts, and excessive deposition of the extracellular matrix (ECM), ultimately leading to irreversible lung architecture destruction and progressive respiratory failure (Martinez et al., 2017; Richeldi et al., 2017). Idiopathic pulmonary fibrosis (IPF) is widely regarded as the most severe subtype of pulmonary fibrosis, characterized by a rapidly progressive disease course, with a median survival of only 3–5 years following diagnosis in many patients, which is substantially shorter than that observed in most secondary forms of pulmonary fibrosis (Lederer and Martinez, 2018; Wijsenbeek et al., 2022). Even with antifibrotic therapy, the majority of patients with IPF exhibit an absolute decline in forced vital capacity (FVC) of ≥5–10% or a reduction in diffusing capacity for carbon monoxide (DLCO) of ≥10–15% within 1 year of follow-up; such progressive deterioration in lung function is strongly associated with an increased risk of mortality or the need for lung transplantation, resulting in an overall mortality rate of 30%–40% or higher over 3–4 years of follow-up (Lee et al., 2024a; Oldham et al., 2025). Despite the availability of antifibrotic agents such as pirfenidone and nintedanib, the therapeutic effect for pulmonary fibrosis is still limited, and lung transplantation is the only curative option, underscoring the need to identify novel pathogenic mechanisms and therapeutic targets (Vancheri et al., 2018).

Emerging evidence highlights dysregulated iron metabolism as a critical contributor to PF. Iron overload promotes reactive oxygen species (ROS) generation, lipid peroxidation, and cellular injury, linking it to ferroptosis-a distinct, iron-dependent mode of programmed cell death (Ali et al., 2017; Ali et al., 2020b; Qiu et al., 2024). Ferroptosis has been increasingly recognized as a driver of epithelial injury, fibroblast activation, and immune dysfunction, thereby accelerating fibrotic remodeling. Within the fibrotic lung microenvironment, complex crosstalk among alveolar epithelial cells, fibroblasts, and macrophages is increasingly recognized to shape ferroptosis-related signaling networks (Ogger and Byrne, 2020; Xu et al., 2021). Despite these advances, several important knowledge gaps remain. First, the precise mechanisms by which ferroptosis contributes to PF initiation and progression are still incompletely understood. Most existing studies focus on isolated molecular pathways or single cell types, providing limited insight into the coordinated, spatiotemporal regulation of ferroptosis across multiple cell populations. Second, ferroptosis is tightly coupled to metabolic reprogramming, including alterations in iron handling, lipid metabolism, and redox homeostasis; however, how these metabolic changes integrate with ferroptotic signaling to drive fibrotic progression has not been systematically examined. Third, although pharmacological modulators of ferroptosis have shown promise in preclinical models, their translational relevance remains uncertain, and the lack of an integrated mechanistic framework hampers rational therapeutic development.

In this review, we summarize recent advances in understanding ferroptosis in PF, with a particular focus on iron dysregulation, lipid peroxidation, and oxidative stress. We highlight emerging evidence linking metabolic reprogramming to ferroptotic vulnerability across key effector cells and discuss the implications of these interactions for disease progression. By integrating these findings, we aim to establish a multidimensional framework that connects metabolism and ferroptosis in PF, thereby clarifying existing knowledge gaps and providing a conceptual basis for future antifibrotic therapeutic strategies.

## 2 Pathological mechanisms of pulmonary fibrosis: the crosstalk between cellular metabolic dysregulation and oxidative stress

**FIGURE 1:**
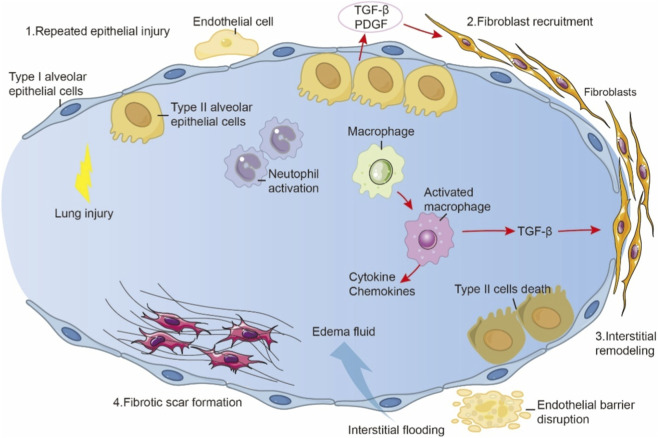
The pathogenic sequence and mechanism driving fibrotic lung disease. Repeated Epithelial Injury: Initial damage to type I alveolar epithelial cells and endothelial cells triggers lung injury, neutrophil activation, and release of profibrotic mediators (TGF-β, PDGF). Fibroblast Recruitment & Macrophage Activation: Inflammatory signals recruit fibroblasts and activate macrophages. TGF-β (from macrophages and epithelial cells) is a major driver of fibroblast activation and proliferation. Cytokines and chemokines amplify the response. Interstitial Remodeling & Vascular Leak: Activated fibroblasts deposit extracellular matrix, causing interstitial remodeling. Endothelial barrier disruption leads to interstitial flooding with edema fluid. Fibrotic Scar Formation & Epithelial Dysfunction: Persistent injury and remodeling result in fibrotic scar formation. Death of type II alveolar epithelial cells impairs repair and surfactant production, further sustaining fibrosis.

The onset of pulmonary fibrosis is typically triggered by chronic or recurrent injury to lung tissue, with damage to alveolar epithelial cells, particularly type II alveolar epithelial cells (AT2 cells), recognized as a critical initiating event. In the early stages of fibrosis, injured AT2 cells release various pro-inflammatory and pro-fibrotic mediators, which contribute to the recruitment, activation, and proliferation of fibroblasts. Activated fibroblasts, in turn, secrete large amounts of collagen and other ECM components to promote tissue repair (Thannickal et al., 2004; Noble et al., 2012). However, when injury persists or repair mechanisms become dysregulated, fibroblasts remain in a sustained state of activation, leading to excessive ECM deposition. This ultimately disrupts alveolar architecture and forms irreversible fibrotic foci (Figure 1). In this process, macrophages and other immune cells especially via their secretion of inflammatory cytokines and chemokines further exacerbate fibroblast activation and ECM accumulation (Shenderov et al., 2021; Moss et al., 2022). It is important to highlight that although these processes are often viewed as components of a “repair response,” they exhibit significant plasticity and dynamic equilibrium. In particular, the interplay between metabolic regulation and oxidative stress frequently determines whether the repair process remains controlled or becomes dysregulated. Recent studies increasingly suggest that cellular metabolic imbalance especially the complex interplay between lipid metabolic reprogramming and oxidative stress acts as a key driver of pulmonary fibrosis. The accumulation of lipid peroxidation products not only damages cellular membrane structures and induces cell death but also influences intercellular signaling pathways, activating fibrosis-associated gene expression (Li et al., 2022b). At the same time, the persistent amplification of oxidative stress exacerbates this cycle of injury and aberrant repair, forming a “metabolism-oxidative stress-fibrosis” positive feedback loop. Notably, this feedback loop is not merely the result of cumulative metabolic disturbances; it involves multilayered regulatory networks encompassing lipid metabolic pathways, redox homeostasis, and cell matrix interactions. These insights provide a rationale for developing multi-targeted intervention strategies in the treatment of pulmonary fibrosis.

Cellular metabolism plays a pivotal role in maintaining cellular function and tissue homeostasis. In the pathogenesis and progression of pulmonary fibrosis, metabolic reprogramming has been recognized as a key hallmark. This reprogramming involves extensive dysregulation across various metabolic processes, including energy metabolism, lipid metabolism, and amino acid metabolism in multiple cell types. Such metabolic disturbances not only directly contribute to fibroblast activation and ECM deposition, but also create favorable conditions for cell death by exacerbating oxidative stress and lipid peroxidation (Rajesh et al., 2023). Type II alveolar epithelial cells play a crucial role in lung tissue repair, as they are responsible for synthesizing pulmonary surfactant and possess stem/progenitor-like capabilities, enabling them to differentiate into type I alveolar epithelial cells and facilitate alveolar reconstruction (Kobayashi et al., 2020). However, within the fibrotic microenvironment, AT2 cells frequently exhibit marked mitochondrial dysfunction, including reduced efficiency of the electron transport chain and diminished ATP synthesis (Rangarajan et al., 2017; Larson-Casey et al., 2020). These mitochondrial impairments not only compromise the cell’s energy supply but also promote excessive ROS generation, amplifying oxidative stress and driving cellular injury and programmed cell death (Larson-Casey et al., 2016; Otoupalova et al., 2020). This oxidative stress imbalance is not only a central cause of the loss of AT2 cell barrier function but also likely serves as a key driver of the persistent progression of pulmonary fibrosis.

During the repair process following lung injury, AT2 cell enhance fatty acid synthesis to support membrane reconstruction and repair functions (Bridges et al., 2010; Olajuyin et al., 2019; Fan et al., 2023). However, excessive activation of fatty acid synthesis coupled with impaired fatty acid oxidation disrupts the normal composition and fluidity of cellular membranes, further compromising surfactant production and undermining alveolar stability and gas exchange function (O'Callaghan et al., 1987; Agudelo et al., 2020; Bargagli et al., 2020). This metabolic imbalance not only promotes ROS generation but also exacerbates lipid metabolic disturbances, creating a self-amplifying cycle of oxidative stress. ROS can trigger lipid peroxidation, leading to the formation of toxic lipid peroxidation products such as 4-hydroxynonenal (4-HNE) and malondialdehyde (MDA), which compromise the integrity and function of cell membranes and ultimately result in cell death (Su et al., 2019; Hirata et al., 2023). Interestingly, these lipid peroxidation products can also act as signaling molecules, activating oxidative stress-related pathways and further inducing the secretion of TGF-β1, thereby reinforcing fibroblast activation and ECM synthesis (Liu and Desai, 2015; Cui et al., 2023; Lee et al., 2024b). Additionally, TGF-β1 can induce epithelial-to-mesenchymal transition (EMT) in AT2 cells, driving them toward a fibroblast-like phenotype that promotes abnormal ECM accumulation and exacerbates pulmonary fibrosis (Bao et al., 2023; Ling et al., 2023). Beyond these processes, lipid peroxidation also interplays with endoplasmic reticulum (ER) stress and immune cell dysfunction, further aggravating the fibrotic microenvironment. The accumulation of lipid peroxidation products can trigger ER stress, promoting AT2 cell apoptosis, EMT processes, and the secretion of pro-fibrotic factors (Geng et al., 2021). These metabolic byproducts can also impair the phagocytic function of alveolar macrophages, fostering chronic inflammation and abnormal fibroblast activation, thereby perpetuating the progression of fibrosis (Minagawa et al., 2020).

In the progression of fibrosis across multiple organs, including the heart, liver, and lungs, dysregulated lipid metabolism and persistent accumulation of ROS synergistically drive the amplification of oxidative stress (Richter et al., 2015; Doridot et al., 2019). Among these processes, lipid peroxidation induced by oxidative stress not only represents a critical mechanism of cellular injury but is also intimately linked to ferroptosis (Galaris et al., 2019). Lipid peroxidation products can react with phospholipids in cell membranes, compromising membrane integrity and inducing membrane damage, which further promotes ROS accumulation in a vicious cycle that ultimately leads to ferroptosis (Ayala et al., 2014). As early as the late 20th century, Rahman and colleagues demonstrated significantly elevated levels of lipid peroxidation products in bronchoalveolar lavage fluid and plasma samples from patients with pulmonary fibrosis, underscoring the importance of lipid peroxidation in this disease (Rahman et al., 1999). Subsequent studies have shown that one of the core lipid peroxidation products, 4-HNE, is markedly upregulated in lung fibroblasts derived from patients with pulmonary fibrosis (Reyes-Jiménez et al., 2021). Additionally, in bleomycin-induced pulmonary fibrosis mouse models, a notable downregulation of GPX4 alongside significant accumulation of 4-HNE has been observed. Notably, pharmacological inhibition of BLM-induced lipid peroxidation has been shown to effectively attenuate experimental pulmonary fibrosis progression, providing critical theoretical support for antifibrotic strategies targeting lipid peroxidation and ferroptosis (Tsubouchi et al., 2019).

Overall, lipid peroxidation emerges as a central driver in the pathogenesis and progression of pulmonary fibrosis: it not only disrupts AT2 cell barrier function and promotes aberrant fibroblast activation thereby sustaining a persistent oxidative injury environment -but also likely interacts with ferroptosis and metabolic reprogramming to collectively fuel a self-amplifying fibrotic cycle. Looking ahead, systematically integrating the dynamic regulation among lipid metabolism, oxidative stress, and cell death signaling, and identifying critical coupling nodes and feedback loops within this network, may offer more precise and innovative therapeutic strategies for pulmonary fibrosis.

## 3 The concept and regulatory mechanisms of ferroptosis

**FIGURE 2:**
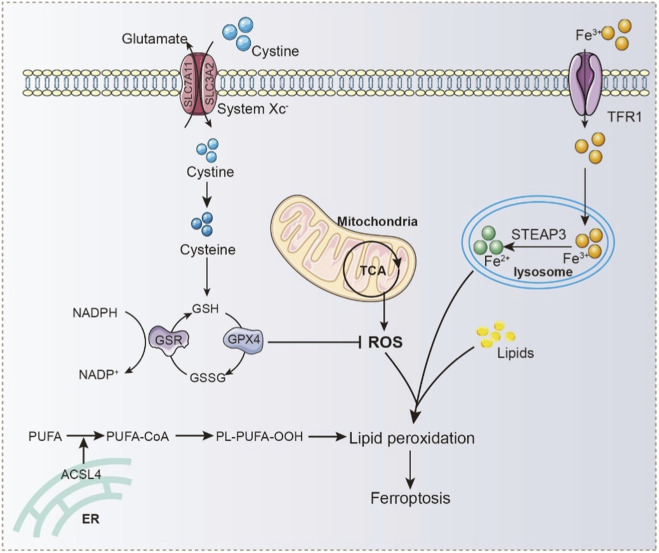
Molecular Network of Ferroptosis: Antioxidant Defense, PUFA Peroxidation, and Iron-Driven Oxidative Stress. Extracellular cystine is imported into the cell via the cystine/glutamate antiporter (system Xc^−^), where it is reduced to cysteine and used to synthesize GSH. GSH serves as a cofactor for GPX4, which reduces lipid hydroperoxides and prevents ferroptosis. NADPH, regenerated through GSR-mediated reduction of GSSG to GSH, maintains redox balance. Disruption of this antioxidant system leads to accumulation of ROS, promoting lipid peroxidation. Concurrently, polyunsaturated fatty acids (PUFAs) are activated by acyl-CoA synthetase long-chain family member 4 (ACSL4) and incorporated into phospholipids, forming PL-PUFA-OOH, which are susceptible to peroxidation. Iron, imported via TFR1 and reduced from Fe^3+^ to Fe^2+^ by STEAP3 in lysosomes, catalyzes lipid peroxidation through the Fenton reaction. ROS generated from mitochondrial metabolism also contributes to oxidative damage.

While the pivotal role of lipid peroxidation in the pathogenesis of pulmonary fibrosis has been widely recognized, increasing evidence suggests that lipid peroxidation is not only a hallmark of oxidative stress damage but also a critical mediator of ferroptosis. Ferroptosis, a form of regulated cell death driven by iron accumulation and lipid peroxidation, has emerged from preclinical studies as a potentially important mechanism contributing to pulmonary fibrosis progression. Its initiation is primarily driven by three interrelated factors: iron accumulation, lipid peroxidation, and dysregulation of antioxidant systems (Figure 2). Unlike traditional forms of cell death such as apoptosis, necrosis, or autophagy, the core mechanism of ferroptosis lies in aberrant intracellular iron overload, which induces oxidative damage to membrane lipids, disrupts membrane integrity and function, and ultimately leads to cell death (Zheng and Conrad, 2020; Dixon and Olzmann, 2024). Iron is an essential trace element involved in various cellular metabolic processes and exists predominantly in two oxidation states: ferrous (Fe^2+^) and ferric (Fe^3+^) iron (Theil, 2004). Under physiological conditions, a tightly regulated iron homeostasis network ensures dynamic equilibrium through coordinated regulation of iron uptake, storage, and export. For instance, transferrin receptor 1 (TFRC) mediates Fe^3+^ uptake, while divalent metal transporter 1 (DMT1) facilitates Fe^2+^ transport into the cytoplasm. Ferritin serves as an intracellular iron storage protein that buffers excess iron and limits its toxicity. Meanwhile, hepcidin and ferroportin (FPN) act together to control iron efflux (Nemeth et al., 2004; Sangkhae and Nemeth, 2017). Disruption of this regulatory network, particularly excessive accumulation of Fe^2+^ can lead to the generation of large amounts of ROS via the Fenton reaction, triggering oxidative stress (Li et al., 2020a; Chen et al., 2021; Lee and Hyun, 2023). The resulting ROS not only damage cellular proteins and DNA but also target polyunsaturated fatty acids (PUFAs) in the plasma membrane, initiating lipid peroxidation reactions. These reactions generate a range of toxic lipid peroxidation products, including MDA and 4-HNE, which compromise membrane structure and function, ultimately culminating in ferroptosis (Liang et al., 2022; Co et al., 2024).

In addition to the core mechanisms described above, multiple signaling pathways and regulatory factors are involved in the progression of ferroptosis. One key component is the system Xc^−^ antiporter, which plays a pivotal role in maintaining intracellular redox homeostasis. Comprising solute carrier family 7 member 11 (SLC7A11) and solute carrier family 3 member 2 (SLC3A2), system Xc^−^ mediates the import of extracellular cystine into the cell. Once inside, cystine is reduced to cysteine, a rate-limiting precursor for the synthesis of glutathione (GSH) (Aboushousha et al., 2023). Inhibition of system Xc^−^ reduces cystine uptake, impairs GSH synthesis, and consequently diminishes the cell’s ability to detoxify lipid peroxides, thereby facilitating the onset of ferroptosis (Bell et al., 2024). The cellular antioxidant defense system is critical for suppressing ferroptosis. Among these, the GSH-glutathione peroxidase 4 (GPX4) axis represents the most well-characterized protective mechanism. GPX4 uses GSH as a substrate to catalyze the reduction of lipid hydroperoxides, thereby protecting membrane integrity from oxidative damage. Ferroptosis is frequently associated with GSH depletion and reduced GPX4 expression or enzymatic activity, both of which compromise the cell’s capacity to neutralize lipid peroxides and promote ferroptosis (Liu et al., 2023; Xue et al., 2023). In recent years, studies have identified additional GPX4-independent pathways that compensate for the loss of GPX4 activity. Notably, ferroptosis suppressor protein 1 (FSP1) and the CoQ10/NADPH system have emerged as crucial regulators. These pathways act through distinct mechanisms to eliminate lipid radicals and maintain cellular redox balance, offering alternative strategies for resisting ferroptosis (Bersuker et al., 2019; Doll et al., 2019).

The occurrence of ferroptosis is not solely driven by disruptions in iron homeostasis, accumulation of lipid peroxides, and dysfunction of the antioxidant defense system. Rather, it is regulated by a complex and finely tuned network involving multiple transcription factors and organelle functions. Among these regulators, nuclear factor erythroid 2-related factor 2 (Nrf2) is one of the most critical transcription factors involved in cellular antioxidant responses. Under basal conditions, Nrf2 is sequestered in the cytoplasm through its interaction with the inhibitory protein Keap1, remaining in an inactive state (Yamamoto et al., 2018). Upon exposure to oxidative stress, Nrf2 dissociates from the Keap1 complex and translocate into the nucleus, where it activates the transcription of a variety of downstream genes related to antioxidant defense and iron metabolism, including SLC7A11, GPX4, and heme oxygenase-1 (HO-1). This response enhances the cellular capacity to eliminate ROS and lipid peroxides, thereby suppressing ferroptosis (Dodson et al., 2019). As such, Nrf2 is regarded as a key protective factor against ferroptosis and a promising therapeutic target in ferroptosis-related pathologies. In contrast, certain stress-related transcription factors act as positive regulators of ferroptosis. For example, activating transcription factor 3 (ATF3), an oxidative stress-inducible transcription factor, has been shown to bind to the promoter region of SLC7A11 under stress conditions, thereby repressing its transcription. This repression reduces cystine uptake, impairs GSH synthesis, and diminishes GPX4 activity, ultimately facilitating lipid peroxidation and inducing ferroptosis (Wang et al., 2020a). Interestingly, another member of the ATF family, ATF4, plays an opposite role. Studies have demonstrated that ATF4 can transcriptionally upregulate SLC7A11 expression, maintain intracellular GSH levels, and suppress ferroptosis-associated necrosis and inflammatory responses (Li et al., 2020a; He et al., 2023). This functional dichotomy within the ATF family suggests a high degree of specialization and regulatory potential in ferroptosis. Additionally, the role of mitochondria in ferroptosis has garnered increasing attention. As the central hub of cellular energy metabolism and redox regulation, mitochondria are directly involved in iron metabolism, ROS production, and lipid peroxidation. It has been reported that ferroptosis is frequently accompanied by mitochondrial membrane potential dissipation, reduced activity of electron transport chain complexes, and the opening of the mitochondrial permeability transition pore (mPTP)-events that promote abnormal ROS release and exacerbate lipid oxidative damage (Gan, 2021). Mitochondrial dysfunction not only reflects the cellular stress state but also serves as an essential mediator in the execution of ferroptosis.

It is noteworthy that the regulatory mechanisms of ferroptosis play critical roles in the pathogenesis of various diseases, including neurodegenerative disorders, cancer, cardiovascular diseases, and pulmonary fibrosis. Iron accumulation exacerbates ROS production via the Fenton reaction and simultaneously promotes lipid peroxidation, leading to disruption of cell membrane structure and loss of function. These processes further accelerate cell death and tissue damage. Therefore, therapeutic strategies aimed at modulating iron homeostasis, enhancing antioxidant defenses, preserving mitochondrial integrity, and inhibiting lipid peroxidation hold great promise for suppressing ferroptosis and mitigating the progression of tissue fibrosis and other pathological conditions.

## 4 Iron metabolism and iron homeostasis

**FIGURE 3:**
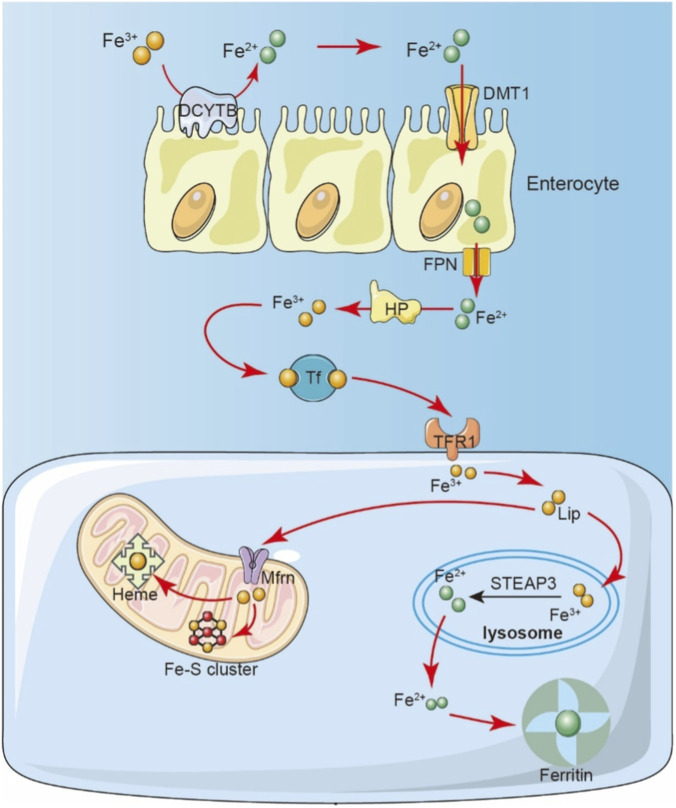
Schematic of mammalian iron absorption, transport, and cellular uptake. Dietary ferric iron (Fe^3+^) is reduced to ferrous iron (Fe^2+^) at the apical surface of enterocytes and imported via the divalent metal transporter DMT1. Intracellular iron (as Fe^2+^) is exported from the enterocyte basolateral membrane into the bloodstream by FPN. It is then oxidized to Fe^3+^ (likely by HP-hephaestin or ceruloplasmin) and bound by serum transferrin (TF) for transport. Transferrin-bound iron (TF-Fe^3+^) is taken up by cells via transferrin receptor 1 into endosomes. Within the acidic endosome/lysosome, STEAP3 reduces Fe^3+^ to Fe^2+^, Ferrous iron (Fe^2+^) is then transported into the cytosol). Iron Utilization: Cytosolic Fe^2+^ is utilized for essential cellular functions, such as the synthesis of iron-sulfur (Fe-S) clusters.

Iron, as an essential trace element for sustaining life, is widely involved in critical physiological processes, including oxygen transport, energy metabolism, DNA synthesis, cell cycle regulation, and redox reactions (Dutt et al., 2022). The body maintains cellular and tissue iron homeostasis through a finely tuned regulatory network that coordinates iron uptake, distribution, storage, utilization, and excretion (Figure 3). Dietary intake represents the primary source of iron, with heme iron being directly absorbed by the intestine, while non-heme iron must first be reduced to ferrous iron by ferric reductases such as DCYTB at the brush border of enterocytes, before being transported into intestinal epithelial cells via divalent metal transporter 1 (DMT1) (Mackenzie and Garrick, 2005; Yanatori and Kishi, 2019). Within these cells, part of the Fe^2+^ is stored in ferritin, while the remainder is exported to the bloodstream by FPN. Once in the blood, Fe^2+^ is oxidized to Fe^3+^ and binds to transferrin (Tf), forming complexes that can be recognized and taken up by iron-demanding tissues, such as the bone marrow (for hematopoiesis), liver (for storage), and spleen (for erythrocyte degradation and iron recycling) (Wang et al., 2020b; Tsyplenkova et al., 2024). Intracellularly, iron not only supports basic metabolic activities but also mediates critical functions within mitochondria, including oxygen transport, electron transfer, and iron-sulfur cluster (Fe-S cluster) protein synthesis (Tang et al., 2021). Disruption of iron homeostasis whether through impaired absorption, abnormal storage, or limited excretion can lead to abnormal iron accumulation in specific tissues, triggering the Fenton reaction to generate ROS. This in turn results in oxidative damage, lipid peroxidation, and cellular dysfunction.

Notably, the human body lacks an active mechanism for iron excretion; iron elimination primarily relies on nonspecific pathways, such as enterocyte shedding, skin desquamation, and iron loss via sweat and urine. Therefore, FPN, currently the only known cellular iron exporter, plays an irreplaceable role in maintaining systemic iron balance. FPN is widely expressed in intestinal epithelial cells, hepatocytes, splenic macrophages, and the placental barrier (Rishi and Subramaniam, 2021). Emerging studies have also demonstrated that in the lung, alveolar epithelial cells, alveolar macrophages, and vascular endothelial cells express FPN, thereby participating in the transmembrane transport and local regulation of iron homeostasis, which in turn significantly influences oxidative stress responses and tissue repair capacity in the lung (Neves et al., 2017). Consequently, systematically elucidating the specific expression and regulatory roles of iron homeostasis-related proteins within the lung is of critical significance for understanding the pathogenesis of diseases such as pulmonary fibrosis, and for providing a theoretical foundation to support potential therapeutic interventions.

## 5 Regulatory mechanisms of iron homeostasis

Iron, as an essential trace element, plays an indispensable role in numerous cellular and systemic physiological processes, including oxygen transport, energy metabolism, DNA synthesis, and cell proliferation. Therefore, the precise regulation of systemic and cellular iron homeostasis is critical to prevent dysfunction resulting from iron deficiency or oxidative damage induced by iron overload. The maintenance of iron homeostasis relies on multi-level regulatory mechanisms that collectively coordinate iron absorption, storage, transport, and utilization, ensuring that intracellular and tissue iron concentrations remain within an optimal range. This regulation is particularly important in oxygen-sensitive organs such as the lungs, where even slight disturbances in iron balance may lead to significant oxidative stress and tissue injury.

Transferrin and the transferrin receptor (TfR) are critical regulators of systemic iron transport. In the plasma, Tf binds to ferric iron (Fe^3+^) to form the Tf-Fe^3+^ complex, which is subsequently recognized by TfR on the surface of cells, particularly those with high iron demand-and internalized via receptor-mediated endocytosis (Dev and Babitt, 2017; Muckenthaler et al., 2017). Expression of TfR correlates with cellular iron requirements, exhibiting a demand-driven regulation of iron uptake. In addition, iron regulatory proteins (IRP1 and IRP2) play a central role in maintaining intracellular iron homeostasis by binding to iron-responsive elements (IREs) located in the untranslated regions of target mRNAs (Zhang et al., 2014; Pasricha et al., 2021). Under conditions of low intracellular iron, IRPs bind to IREs to stabilize TfR mRNA, thereby enhancing TfR expression and promoting iron uptake. Concurrently, IRP binding suppresses the translation of ferritin mRNA, reducing iron storage and prioritizing the availability of functional iron. Conversely, when intracellular iron levels are elevated, IRPs dissociate from IREs, leading to decreased TfR expression and increased ferritin synthesis, thereby facilitating iron sequestration and detoxification (Muckenthaler et al., 2017; Galy et al., 2024).

Moreover, when intracellular iron levels rise, the Homeostatic Iron Regulator (HFE) protein binds to TfR1, thereby reducing its affinity for iron-loaded transferrin (Fe-Tf) and limiting further cellular iron uptake (Billesbølle et al., 2020). This mechanism is essential for sensing systemic iron status and initiating appropriate regulatory responses. Mutations in the HFE gene are the major cause of hereditary hemochromatosis, a genetic disorder characterized by impaired regulation of TfR-mediated iron uptake, leading to excessive iron absorption and iron overload in organs such as the liver, heart, pancreas, and joints, ultimately resulting in tissue damage (Brissot et al., 2018; Mottelson et al., 2024). Importantly, HFE also acts as an upstream regulator of hepcidin expression. Hepcidin, a liver-derived peptide hormone, serves as the central regulator of systemic iron homeostasis. Its expression is modulated by multiple signals, including serum iron levels, intracellular iron status, inflammatory cytokines, and the bone morphogenetic protein (BMP)/SMAD signaling pathway (Yang et al., 2020; Sardo et al., 2024). In response to increased iron load or inflammatory stimuli, hepcidin expression is upregulated. Hepcidin binds to FPN, the only known cellular iron exporter, inducing its ubiquitination and subsequent degradation, thereby blocking iron efflux from enterocytes and macrophages and reducing circulating iron levels (Camaschella, 2013; Camaschella et al., 2020). However, studies have shown that hepcidin expression is relatively low in lung tissues, particularly in alveolar epithelial cells and alveolar macrophages, suggesting that its role in the lung may be predominantly paracrine or systemic rather than cell-autonomous (Chen et al., 2014). Dysregulation or deficiency of hepcidin has been identified as a major mechanism underlying both systemic and local iron overload, contributing to chronic injury in multiple organs (Billesbølle et al., 2020; Nemeth and Ganz, 2023). In contrast, ferritin, the primary intracellular iron storage protein, acts as a crucial buffer in maintaining iron homeostasis. It safely sequesters excess intracellular Fe^2+^ in a non-toxic form, preventing it from catalyzing the Fenton reaction and generating ROS, thereby protecting tissues from oxidative damage (Arosio et al., 2017). Under iron overload conditions, ferritin synthesis is markedly upregulated, particularly in hepatocytes, macrophages, and alveolar epithelial cells, ensuring both safe iron storage and bioavailability (Shesh and Connor, 2023; Liu et al., 2024b). In addition, alveolar macrophages, as key regulators of pulmonary iron metabolism, play an essential role in recycling iron from apoptotic cells and erythrocyte remnants. They can phagocytose these materials, recover the iron content, and regulate its release to prevent local iron deposition, thereby preserving pulmonary iron homeostasis (Ludwig et al., 2024).

In summary, alveolar epithelial cells, alveolar macrophages, and vascular endothelial cells within the lung express essential iron-handling proteins such as TfR, ferritin, FPN, and their regulatory transcription factors. These components form a coordinated regulatory network that maintains optimal iron levels both systemically and in the lung microenvironment. Disruption of this network may lead to systemic iron overload and contribute to pathological processes such as oxidative stress, inflammation, and ferroptosis, ultimately promoting lung injury and the progression of pulmonary fibrosis.

## 6 Disruption of pulmonary iron homeostasis and ferroptosis

Iron plays a critical role in maintaining normal pulmonary physiological functions, being widely involved in oxygen transport, cellular energy metabolism, and antioxidant defense. Under normal physiological conditions, lung tissue primarily acquires iron through the bloodstream, while iron inhaled directly via the respiratory tract accounts for only a minimal fraction. However, due to the lung’s direct exposure to the external environment, alveolar epithelial cells are chronically challenged by exogenous factors such as iron-containing particulate matter and pathogenic microorganisms, which can disrupt the dynamic balance of iron metabolism (Suresh and Shimoda, 2016). Various external environmental factors-including cigarette smoking, air pollution, and occupational dust exposure-can increase iron deposition within the respiratory tract, thereby elevating local iron load and promoting or exacerbating inflammatory responses (Zhang et al., 2019c). Additionally, bacterial infections (e.g., *Pseudomonas aeruginosa* or *Legionella pneumophila*) can secrete high-affinity iron chelators (siderophores) that compete with host cells for intracellular iron, disrupting host iron homeostasis and facilitating bacterial colonization and pathogenicity in lung tissue (Cianciotto, 2015; Lamberti and Surmann, 2021). Pulmonary hemorrhage can further exacerbate pulmonary iron overload by releasing hemoglobin and free iron through erythrocyte lysis, increasing oxidative stress risk (Wang and Babitt, 2019). These exogenous and endogenous factors act synergistically, potentially causing severe disruption of iron homeostasis in lung tissue. This disruption not only impairs normal cellular functions but may also trigger ferroptosis, mitochondrial dysfunction, and lipid peroxidation of cell membranes, culminating in chronic inflammation, tissue injury, and the initiation and progression of pulmonary fibrosis.

To maintain iron homeostasis, the lung relies on a series of finely tuned and complex regulatory mechanisms involving iron uptake, storage, utilization, and export. Under physiological conditions, alveolar epithelial cells and alveolar macrophages mediate iron uptake primarily through the expression of DMT1, while hepcidin binds to FPN, inducing its ubiquitination and degradation, thereby inhibiting transmembrane iron export and controlling systemic iron availability (Nemeth and Ganz, 2021). In addition, alveolar lining fluid contains various iron-binding proteins, such as lactoferrin, ferritin, and transferrin, which chelate free iron and limit its participation in the Fenton reaction, thus mitigating oxidative stress-induced damage to lung tissue (Kaczyńska et al., 2023; Zhang et al., 2025). Alveolar macrophages also play a crucial role in regulating iron homeostasis by phagocytosing leaked erythrocytes and degrading heme iron, thereby effectively eliminating excess iron and preventing its abnormal accumulation (Drakesmith et al., 2015). However, under various pathological conditions-including smoking, air pollution, chronic inflammation, and pulmonary infections-these regulatory mechanisms are often disrupted, leading to enhanced iron uptake, impaired storage capacity, and hindered iron export, ultimately resulting in abnormal pulmonary iron accumulation. During the onset and progression of pulmonary fibrosis, increased iron burden has been increasingly recognized as an important factor implicated in pathological remodeling. Excess free iron can continuously generate ROS via the Fenton reaction, inducing lipid peroxidation, DNA damage, and protein denaturation, thereby contributing to structural and functional impairment of lung tissue (Turi et al., 2004). Iron-mediated oxidative stress not only damages alveolar epithelial cells but also triggers ferroptosis in fibroblasts and macrophages, further exacerbating inflammation, cell death, and extracellular matrix deposition, and ultimately promoting the persistent progression of fibrosis.

Iron-mediated oxidative stress is recognized as a critical triggering mechanism in acute lung injury (ALI) and various chronic pulmonary diseases. Studies have shown that patients with cystic fibrosis frequently exhibit dysregulated iron metabolism, with aberrant iron accumulation creating a favorable environment for microbial pathogens-particularly *P. aeruginosa* thereby exacerbating infection and inflammatory responses and accelerating disease progression (Reid et al., 2002; Jia et al., 2024). Similarly, disruption of iron homeostasis has been implicated in the pathogenesis of numerous pulmonary conditions, including COPD, asthma, silicosis, and lung cancer (Zhang et al., 2019b). Under iron overload conditions, excess free iron catalyzes the generation of ROS through the Fenton reaction, leading to pronounced oxidative stress and promoting lipid peroxidation, which in turn triggers ferroptosis-a regulated, iron-dependent form of cell death. Chronic pulmonary inflammation further exacerbates iron dyshomeostasis, resulting in increased iron accumulation in alveolar epithelial cells and macrophages, thereby forming a vicious cycle (Ghio et al., 2006). Concurrently, iron overload suppresses the activity of the cystine/glutamate antiporter system Xc^−^, reduces intracellular GSH levels, and inhibits the activity of GPX4, collectively weakening the cell’s antioxidant defense (Wang et al., 2022b). This redox imbalance ultimately leads to severe damage to membrane lipids, dysfunction and death of alveolar epithelial and vascular endothelial cells, and consequent lung tissue injury and respiratory impairment.

## 7 The impact of ferroptosis on pulmonary fibrosis

Ferroptosis is a form of regulated cell death characterized by iron-dependent lipid peroxidation. The central mechanism involves the catalytic activity of ferrous iron (Fe^2+^) or lipoxygenases (LOXs) on PUFAs within membrane phospholipids, leading to the formation of lipid peroxides, disruption of membrane integrity, and ultimately, cell death (Chen et al., 2020; Li and Li, 2020). In diseases such as IPF, ferroptosis has been increasingly recognized as an important pathological mechanism involved in disease progression. It is well established that oxidative stress plays a critical role in alveolar epithelial injury and fibrogenesis in IPF. Under conditions of chronic inflammation and sustained oxidative stress, pulmonary iron homeostasis becomes dysregulated, resulting in abnormal accumulation of iron ions. Notably, many redox-active enzymes require transition metals, especially iron, for their function. Iron overload accelerates lipid peroxidation through the Fenton reaction, inducing ferroptosis in alveolar epithelial cells, which further exacerbates alveolar destruction and fibrotic tissue remodeling. This suggests a deeper regulatory role for iron in lung pathophysiology (Bargagli et al., 2017). Moreover, the execution of ferroptosis is closely linked to GSH depletion and inactivation of GPX4. When the cellular antioxidant defense is compromised, GPX4 fails to eliminate lipid peroxides, allowing ferroptosis to proceed (Dixon and Pratt, 2023). Recent studies in multiple murine models of pulmonary fibrosis have demonstrated increased pulmonary iron deposition and aberrant expression of ferroptosis-related genes, including GPX4 and SLC7A11, further substantiating the pivotal role of ferroptosis in the pathogenesis of fibrosis. Importantly, interventions aimed at upregulating GPX4 or SLC7A11 to enhance intracellular GSH levels have been shown to effectively inhibit ferroptosis, thereby attenuating lung tissue damage and fibrotic progression (Liu et al., 2024a; Zhang et al., 2024b). These findings provide a theoretical basis for ferroptosis as a therapeutic target in pulmonary fibrosis and suggest that targeting the ferroptotic pathway may represent a novel strategy for clinical intervention.

**FIGURE 4:**
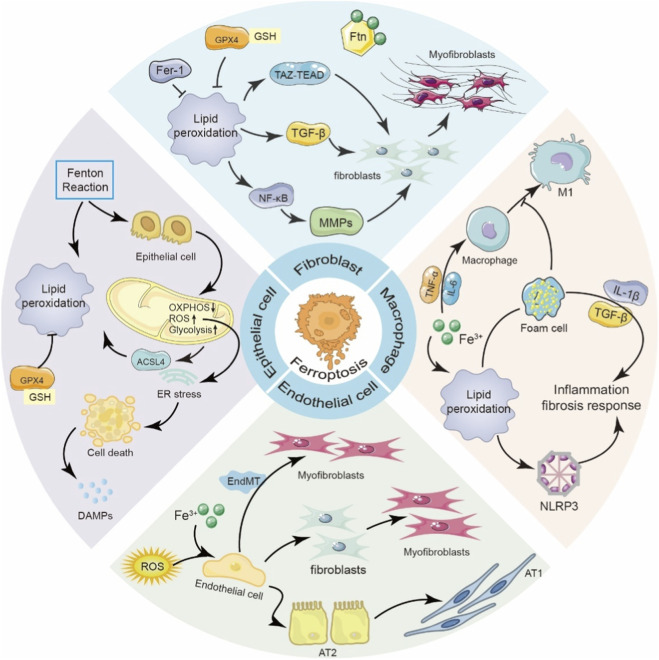
Ferroptosis contributes to pulmonary fibrosis through cell-type-specific mechanisms. This schematic illustrates the roles and mechanisms of ferroptosis in various cell types involved in pulmonary fibrosis, including epithelial cells, fibroblasts, macrophages, and endothelial cells. In alveolar epithelial cells, iron overload and lipid peroxidation drive oxidative stress, ER stress, and ferroptosis, leading to the release of DAMPs and promoting fibroblast activation. In fibroblasts, ferroptosis and associated lipid peroxidation activate TGF-β, TAZ-TEAD signaling, and matrix metalloproteinases, promoting myofibroblast differentiation and ECM deposition. In macrophages, iron accumulation and foam cell formation drive inflammatory responses and fibrosis via IL-1β, TGF-β, and the NLRP3 inflammasome. In endothelial cells, ferroptosis and ROS production disrupt endothelial barrier integrity and induce endothelial-to-mesenchymal transition (EndMT), contributing to myofibroblast expansion.

Studies have demonstrated that in both patients with IPF and animal models of pulmonary fibrosis, extracellular free iron levels and the expression of several iron metabolism-related proteins-such as ferritin and transferrin receptor-are significantly upregulated in lung tissues, suggesting that iron homeostasis disruption may be a major driver of fibrotic remodeling (Wu et al., 1989). In an iron-overloaded environment, alveolar epithelial cells are particularly susceptible to oxidative stress-induced damage, which can trigger ferroptosis, compromise epithelial barrier integrity, and promote inflammatory cell infiltration and aberrant fibroblast activation, thereby accelerating fibrotic progression (Pei et al., 2022). In bleomycin-induced mouse models of pulmonary fibrosis, elevated levels of free iron and increased expression of ferritin have been observed in both alveolar macrophages and fibroblasts, further supporting a close link between iron dysregulation and lung fibrosis (Zhu et al., 2021). Notably, iron accumulation is associated not only with airway remodeling and pulmonary function decline but also with the abnormal proliferation of human lung fibroblasts. This is accompanied by the release of proinflammatory cytokines such as interleukin-6 (IL-6) and tumor necrosis factor-alpha (TNF-α), as well as excessive deposition of ECM components, including collagen and fibronectin, contributing to the establishment of a pro-fibrotic microenvironment (Ali et al., 2020a). Collectively, these findings indicate that Ferroptosis plays a pivotal role in the initiation and progression of pulmonary fibrosis by orchestrating multiple pathological processes, including the activation and proliferation of fibroblasts, injury to alveolar epithelial cells, and the modulation of macrophage polarization (Figure 4).

### 7.1 Ferroptosis induces epithelial cell death

Studies have shown that dysregulation of iron homeostasis in alveolar epithelial cells (AECs) plays a critical role in the onset and progression of pulmonary fibrosis. Under normal physiological conditions, AECs maintain the structural integrity of alveoli, participate in gas exchange, and regulate the local microenvironment through the secretion of surfactants and cytokines (Wang et al., 2023). However, during the fibrotic process, AECs are subjected to persistent injury, leading to aberrant activation of various forms of programmed cell death, including apoptosis, necrosis, and ferroptosis, which act as key drivers of disease progression (Sauler et al., 2019; Parimon et al., 2020; Yao et al., 2021). The death of AECs not only compromises the alveolar barrier, resulting in alveolar collapse and impaired gas exchange, but also promotes the release of pro-inflammatory mediators and cellular debris, which further activate alveolar macrophages and fibroblasts, exacerbating local inflammation and fibrotic remodeling (Hou et al., 2021). In patients with IPF and in bleomycin-induced mouse models of lung fibrosis, abnormal iron accumulation has been observed within AECs, suggesting that iron overload-induced ferroptosis may be a central mechanism contributing to fibrogenesis. Treatment with deferoxamine, an iron chelator, significantly reduces iron accumulation and ferroptosis in type II alveolar epithelial cells, thereby attenuating bleomycin-induced pulmonary fibrosis in mice (Cheng et al., 2021a). Moreover, GPX4 and FSP1 synergistically regulate ferroptosis in AT2. Recent studies have found that the methylation regulator UHRF1 is markedly upregulated in fibrotic lungs and promotes ferroptosis in AT2 by epigenetically suppressing the expression of GPX4 and FSP1. Targeted inhibition of UHRF1 effectively interrupts this pathological process, significantly reduces ferroptosis, and ameliorates fibrotic progression (Liu et al., 2022c). Collectively, these findings underscore the central pathological role of AT2 ferroptosis in pulmonary fibrosis and suggest that targeting iron homeostasis and ferroptosis related signaling pathways in AECs may represent a promising therapeutic strategy for clinical intervention in this disease.

Mitochondrial injury directly impairs the function of alveolar epithelial cells, thereby triggering local inflammatory responses and promoting fibroblast activation driven by TGF-β, a process that plays a critical role in the development of pulmonary fibrosis (Liu and Chen, 2017; Louzada et al., 2021). Damage to AECs not only compromises the alveolar barrier but also leads to mitochondrial dysfunction, which disrupts oxidative phosphorylation and concurrently enhances glycolytic flux (Bueno et al., 2020; Katzen and Beers, 2020). The upregulation of glycolysis results in increased intracellular lactate levels, which in turn induces the expression of ACSL4, a key regulator of ferroptosis. This promotes ferroptosis AECs and exacerbates lung injury (Wu et al., 2024a). Moreover, mitochondrial iron accumulation disrupts mitochondrial function, leading to abnormal cell metabolism. This injury also boosts ROS production and damages mitochondrial DNA, worsening cellular injury (Patnaik and Tefferi, 2019; Li et al., 2020b). Therefore, mitochondrial iron accumulation in AT2 cells may represent a crucial factor driving epithelial damage and pulmonary fibrosis. Restoration of mitochondrial function has been shown to attenuate mitochondrial iron overload and cellular injury in AT2 in bleomycin-induced murine models, thereby effectively suppressing fibrotic progression (Shao et al., 2024). As a major inhibitor of ferroptosis, GPX4 exhibits markedly reduced expression and activity during pulmonary fibrosis, particularly in type II alveolar epithelial cells. The diminished activity of GPX4 disrupts the cellular antioxidant defense system and exacerbates mitochondrial damage, thereby accelerating ferroptosis. Use of ferroptosis inhibitors can restore antioxidant capacity and enhance GPX4 activity, which mitigates lipid peroxidation and reduces lung injury and fibrosis (Zhang et al., 2024b). Furthermore, recent studies have demonstrated that bisphenol A (BPA) induces pulmonary fibrosis in mice by activating the AMPK/mTOR signaling pathway, which promotes autophagy-mediated ferroptosis. Notably, inhibition of autophagy significantly alleviates BPA-induced injury and effectively attenuates the progression of pulmonary fibrosis (Liang et al., 2024).

The core mechanism of ferroptosis involves iron-dependent lipid peroxidation, in which the endoplasmic reticulum-a critical organelle for lipid biosynthesis and protein folding-plays a pivotal role. During ferroptosis, the accumulation of lipid peroxidation products disrupts the structural integrity of the ER membrane, thereby inducing ER stress. ER stress has been widely recognized as an important contributor to the initiation and progression of pulmonary fibrosis (Kropski and Blackwell, 2018). Studies have shown that key regulators of ER stress, such as C/EBP homologous protein (CHOP) and members of the activating transcription factor family, are abnormally expressed during ferroptosis, further underscoring the close relationship between ER stress and ferroptosis (Wang et al., 2020a; Yang et al., 2022). Moreover, ROS-induced ER stress significantly promotes epithelial ferroptosis, resulting in alveolar structural damage, infiltration of inflammatory cells, and decline in lung function (Li et al., 2022c). Further investigations have demonstrated that alleviating ER stress can effectively suppress ferroptosis, thereby reducing alveolar epithelial cell injury and restoring pulmonary function in murine models (Jiang et al., 2023).

Fibrosis, at its core, is a protective reparative response following tissue injury; however, under certain conditions, this process may become dysregulated, resulting in irreversible tissue remodeling and eventual organ failure. In recent years, senescent type II alveolar epithelial cells have emerged as important drivers of pulmonary fibrosis due to their unique senescence-associated secretory phenotype (SASP) (Yao et al., 2021; Han et al., 2023). It has been reported that senescent cells across various fibrotic diseases exhibit marked intracellular iron accumulation (Killilea et al., 2003; Wu et al., 2024b). Notably, in bleomycin-induced pulmonary fibrosis mouse models, significant iron deposition is observed in the trachea, and intratracheal administration of iron exacerbates epithelial senescence and accelerates fibrotic progression (Maus et al., 2023). Importantly, senescent cells resulting from fibrotic injury continue to accumulate iron even after extracellular iron levels return to normal, primarily storing iron in the form of ferritin-bound iron within lysosome (Masaldan et al., 2018). These cells maintain elevated levels of labile iron, which enhances ROS production and amplifies SASP-mediated inflammatory and profibrotic responses, ultimately aggravating tissue damage and promoting fibrotic progression.

In addition to their essential role in gas exchange, alveolar epithelial cells undergoing ferroptosis also contribute to the release of various profibrotic factors and damage-associated molecular patterns (DAMPs). These molecules not only induce the proliferation and differentiation of mesenchymal fibroblasts, but ultimately promote the development of pulmonary fibrosis (Bale et al., 2023). In particular, during ferroptosis, AECs collapse due to iron overload and lipid peroxidation-induced damage, releasing large amounts of ROS, which further activate fibroblasts and enhance ECM deposition, thereby accelerating pulmonary tissue stiffening (Cheng et al., 2021a). Consequently, therapeutic strategies targeting iron metabolism-such as local or systemic administration of iron chelators, or activation of antioxidant pathways-can effectively reduce labile iron accumulation, alleviate oxidative stress, and restore mitochondrial function in epithelial cells. These approaches offer promising new directions for the treatment of pulmonary diseases. Overall, the interplay of lipid metabolic dysregulation, lipid peroxidation, and oxidative stress is critically important in AT2 cell injury and in the initiation and progression of pulmonary fibrosis. These interactions not only exacerbate cellular dysfunction but also contribute to the impairment of alveolar function, promotion of inflammatory responses, and the release of profibrotic mediators, collectively accelerating fibrotic progression. A deeper understanding of these mechanisms and their interconnections not only provides important insights for the prevention and treatment of pulmonary fibrosis but also reveals potential therapeutic targets for future clinical intervention.

### 7.2 Ferroptosis regulates differentiation of fibroblast

During the development of pulmonary fibrosis, fibroblasts undergo aberrant activation and excessive proliferation driven by various cytokines, leading to the accumulation of large amounts of ECM, which in turn causes scarring and stiffening of lung tissue. Simultaneously, interactions between fibroblasts and the local immune microenvironment form a profibrotic positive feedback loop (Kamiya et al., 2024). Moreover, persistently activated fibroblasts frequently exhibit metabolic reprogramming, which further stabilizes and reinforces their pathological phenotype, thereby accelerating fibrotic progression (El Agha and Thannickal, 2023). Thus, fibroblasts not only serve as the central effector cells in pulmonary fibrosis but also represent key therapeutic targets in current antifibrotic strategies.

TGF-β/Smad signaling pathway is one of the classical pathways regulating pulmonary fibrosis and plays a pivotal role in the differentiation of fibroblasts into myofibroblasts. Studies have shown that lipid peroxidation products and ROS which are associated with ferroptosis, exert critical regulatory effects on this process. Specifically, elevated intracellular lipid peroxidation levels enhance TGF-β-mediated activation of Smad2/3 signaling, thereby promoting the trans differentiation of fibroblasts into a myofibroblast phenotype (Tsubouchi et al., 2019). Further investigations have revealed that the ferroptosis inducer erastin exacerbates pulmonary fibrosis by increasing lipid peroxidation and suppressing GPX4 expression, thereby amplifying TGF-β1-induced fibroblast activation (Gong et al., 2019). In contrast, ferroptosis inhibitors markedly attenuate this process by suppressing lipid peroxidation, restoring GPX4 expression, and downregulating TGF-β1 signaling, ultimately mitigating the progression of experimental pulmonary fibrosis (Li et al., 2019; Zhang et al., 2024a). These findings suggest that GPX4, acting as a key regulatory node linking ferroptosis and TGF-β signaling, may be a promising therapeutic target for pulmonary fibrosis. Notably, sustained activation of TGF-β1 also enhances the expression of TFRC in fibroblasts via the TAZ-TEAD signaling axis, leading to increased intracellular Fe^2+^ levels, which in turn promotes fibroblast activation and differentiation. Animal studies have further confirmed that TFRC deletion in mice significantly suppresses TGF-β1-induced Fe^2+^ accumulation and fibroblast activation, and effectively alleviates bleomycin-induced fibrotic changes in the lungs (Pei et al., 2022).

Zhu et al. reported significant iron accumulation in the lung tissues of patients with IPF, accompanied by markedly elevated transcriptional levels of ferritin light chain (Ferritin-L) in pulmonary fibroblasts. These findings suggest that dysregulation of iron homeostasis may be involved in the pathogenesis of IPF. Further studies demonstrated that the iron chelator clioquinol effectively inhibits fibroblast activation, including cell proliferation, myofibroblast differentiation, pro-inflammatory cytokine secretion, and migratory capacity, thereby attenuating bleomycin-induced pulmonary fibrosis in mice (Zhu et al., 2021). Interestingly, ferritin, the primary intracellular iron storage protein, plays a protective role by sequestering labile iron, thereby limiting hydroxyl radical production via the Fenton reaction and preventing ferroptosis (Zhang et al., 2021). This mechanism may explain why iron accumulation during fibrosis does not induce fibroblast death but rather contributes to their activation. Moreover, studies have shown that moderate levels of lipid peroxidation may not necessarily trigger cell death; instead, they can initiate protective stress responses such as the upregulation of heat shock proteins (HSPs) and anti-apoptotic proteins, which enhance fibroblast survival and resistance to external insults (Zhu et al., 2017b). These findings suggest that in the fibrotic lung microenvironment, adaptive changes in iron metabolism and lipid peroxidation may work synergistically to promote the pathological activation of fibroblasts rather than their death.

Moreover, lipid peroxidation products generated during ferroptosis do not always directly induce cell death; some of these metabolites may even exert pro-fibrotic effects under specific pathological conditions. For instance, 4-HNE, a typical end product of lipid peroxidation, has been shown to mediate TGF-β1-induced differentiation of fibroblasts into myofibroblasts. Additionally, 4-HNE can activate the NF-κB signaling pathway, promote the expression of multiple pro-fibrotic factors, enhance cell migratory capacity, and regulate the expression of matrix metalloproteinases (MMPs) *in vitro*, thereby accelerating interstitial remodeling and fibrogenesis in the lung (Xu et al., 2017; Zhao et al., 2024). Ferroptosis stimuli also affect the migratory and proliferative capacity of pulmonary fibroblasts, further exacerbating tissue stiffness and structural disruption in the lung (Li et al., 2024a). Within the fibrotic lung microenvironment, the accumulation of iron promotes the Fenton reaction, leading to the sustained amplification of lipid peroxidation and establishing a pathological state of “high oxidative stress-high adaptability” in fibroblasts. This condition supports their persistent activation and enhances their fibrotic phenotype. Furthermore, aberrant lipid peroxidation not only promotes fibroblast activation via stress response pathways but may also drive continuous ECM synthesis and deposition by reprogramming cellular metabolic pathways, such as mitochondrial function, fatty acid oxidation, and glycolysis. This process plays a central role in the progression of pulmonary fibrosis, highlighting the regulatory interplay between lipid metabolism and ferroptosis as a potential therapeutic target for future anti-fibrotic strategies.

Lipid peroxidation is not only a key marker of cellular injury in pulmonary fibrosis but also serves as a central driver of fibroblast activation and the ensuing fibrotic cascade. Ferroptosis, as a form of cellular stress response, does not invariably culminate in cell death; particularly in fibroblasts, it often manifests as a sub-lethal stress state characterized by enhanced differentiation and activation. In this state, fibroblasts exhibit marked “adaptive survival” capabilities, enabling them to evade death within the fibrotic lung microenvironment and instead acquire heightened fibrogenic potential through ferroptosis-related signaling pathways. According to existing studies, fibroblasts are highly likely to compensate for the inactivation of the intracellular GPX4-mediated antioxidant pathway by upregulating the FSP1-CoQ10 compensatory axis to strengthen their antioxidant defenses. Ferroptosis Suppressor Protein 1 (FSP1), as a GPX4-independent ferroptosis suppressor, utilizes NAD(P)H to reduce oxidized coenzyme Q10 (CoQ10) to its reduced form (CoQ10H_2_), effectively interrupting the chain reaction of lipid peroxidation in cellular membranes (Doll et al., 2019; Nakamura et al., 2023). In the high oxidative stress microenvironment of the lung, the FSP1-CoQ10 pathway not only protects fibroblasts from ferroptosis-induced membrane rupture and functional impairment, but may also promote their survival and persistent activation within the fibrotic niche, forming an “adaptive barrier” that sustains fibrogenesis.

Moreover, in the fibrotic microenvironment characterized by a surge of cytokines and growth factors, activation of this pathway may provide essential conditions for the continuous proliferation and differentiation of fibroblasts, thereby supporting excessive extracellular matrix deposition and pulmonary tissue remodeling. Intriguingly, persistent activation of the FSP1-CoQ10 axis may not merely be a protective adaptation; it may also grant fibroblasts metabolic advantages by sustaining mitochondrial metabolism under high oxidative stress, thereby mitigating ferroptosis-driven damage (Deshwal et al., 2023). Consequently, upregulation of the FSP1-CoQ10 axis in fibroblasts may serve as a critical safeguard for their fibrogenic adaptation and points to the potential of targeting this compensatory mechanism as a novel therapeutic strategy to alleviate pulmonary fibrosis. These observations also suggest that simply inhibiting lipid peroxidation or ferroptosis may be insufficient to fully suppress the aberrant activation of fibroblasts. Future studies should further explore whether there exist more complex signal couplings and dynamic crosstalk between ferroptosis, oxidative stress adaptation, and metabolic reprogramming in this context, in order to identify breakthrough points for therapeutic intervention in pulmonary fibrosis.

### 7.3 Ferroptosis mediates macrophage polarization and foam cell formation

The immune-inflammatory response is a continuous and integral component throughout the pathogenesis of pulmonary fibrosis, serving as a crucial bridge linking external stimuli to tissue injury. As one of the most abundant immune cell populations in lung tissue, macrophages play multiple pivotal roles within the fibrotic microenvironment. Beyond their involvement in pathogen clearance and maintenance of tissue homeostasis, macrophages directly contribute to fibroblast activation and ECM deposition by secreting cytokines and modulating the local immune milieu, thereby regulating the progression of pulmonary fibrosis (Cheng et al., 2021b).

Macrophages influence pulmonary fibrosis through various mechanisms, participating in immune responses as well as modulating fibrotic processes via metabolic reprogramming. Importantly, the metabolic pathways of macrophages decisively determine their polarization state during the course of disease (Thibaut et al., 2022). In the early stages of fibrosis, macrophages polarize toward the M1 phenotype in response to stimuli such as IFN-γ and LPS. M1 macrophages predominantly secrete pro-inflammatory factors including TNF-α, IL-1β, IL-6, and nitric oxide, playing essential roles in pathogen defense, clearance of damaged tissue, and initiation of inflammatory responses. However, prolonged activation of M1 macrophages can provoke excessive inflammation and exacerbate alveolar epithelial cell injury (Yang et al., 2023). During tissue repair, macrophages shift toward the M2 phenotype to resolve inflammation and promote tissue regeneration. Notably, M2 macrophages are major sources of TGF-β1 and platelet-derived growth factor (PDGF), which induce fibroblast differentiation into myofibroblasts, enhance collagen deposition, and facilitate ECM remodeling, thereby accelerating the progression of pulmonary fibrosis (Wang et al., 2021b). Therefore, the dynamic balance and interconversion between M1 and M2 macrophages critically determine the trajectory and severity of pulmonary fibrosis.

In pulmonary fibrosis research, accumulating evidence indicates that macrophages in fibrotic lungs exhibit reduced sensitivity to ferroptosis; however, they can undergo functional changes by sensing ferroptosis-related metabolic signals. Although the accumulation of iron ions and lipid peroxides does not directly induce macrophage death, it may alter their polarization states, thereby modulating their roles during fibrosis. Studies have shown that iron overload promotes macrophage polarization toward the pro-inflammatory M1 phenotype, accompanied by significant upregulation of cytokines such as IL-6, TNF-α, and IL-1β (Zhou et al., 2018; Handa et al., 2019). In cardiovascular disease models, iron accumulation enhances glycolytic metabolism in macrophages, further facilitating M1 polarization, which implies a close relationship between iron metabolic status and macrophage function (Hu et al., 2019). However, macrophage polarization is not exclusively skewed toward M1. Particularly under conditions of lipid peroxidation and iron overload, macrophages are prone to polarize toward the M2 phenotype. M2 macrophages maintain their tissue repair and anti-inflammatory functions by activating fatty acid synthesis and storage pathways, playing crucial roles in chronic inflammation and fibrosis progression (Yan and Horng, 2020; Vassiliou and Farias-Pereira, 2023). Moreover, cytokines and growth factors secreted by M2 macrophages not only promote myofibroblast differentiation and angiogenesis but also exacerbate pulmonary fibrosis by inducing EMT (Zhu et al., 2017a). These findings suggest that iron overload disrupts the balance between M1 and M2 macrophage polarization and plays a vital role in the transition from the inflammatory to the tissue repair phase in pulmonary fibrosis. By modulating macrophage polarization, iron metabolism influences the magnitude and direction of immune responses and may contribute to the progression of pulmonary fibrosis. Therefore, targeting the interplay between iron metabolism and macrophage polarization offers promising therapeutic strategies for pulmonary fibrosis.

During the development of pulmonary fibrosis, macrophages play a critical role in the early clearance of lipids in the lung. However, as fibrosis progresses, dysregulated lipid metabolism significantly modulates macrophage function, particularly influencing lipid peroxidation and foam cell formation. Excessive lipid accumulation, especially the peroxidation of polyunsaturated fatty acids, leads macrophages to engulf large amounts of lipids, resulting in foam cell formation and subsequent functional impairment (Li et al., 2022a). Moreover, lipid peroxidation products not only exacerbate ROS generation by directly damaging cellular membranes but also activate inflammasomes such as NLRP3, thereby sustaining and amplifying inflammatory responses (Huang et al., 2021). In bleomycin-induced pulmonary fibrosis models, macrophages respond to lipid accumulation by dumping lipids into the distal airspaces of the lung. Nevertheless, under pathological conditions, apolipoprotein deficiency or excessive lipid accumulation can promote cholesterol buildup within alveolar macrophages, inducing foam cell formation (Romero et al., 2015; Wygrecka et al., 2023). The accumulation and death of these foam cells release substantial amounts of pro-inflammatory and pro-fibrotic factors, intensifying chronic inflammation and fibroblast activation, which exacerbate fibrosis progression. Interestingly, foam cells appear to influence macrophage polarization as well. *In vitro* induction of foam cell formation not only alters macrophage metabolic states but may also suppress M1 polarization, attenuating pro-inflammatory capacity and skewing macrophages toward an immunosuppressive or reparative phenotype (da Silva et al., 2016). This polarization shift may partly regulate the transition from inflammation to fibrosis in pulmonary fibrosis, highlighting the dual role of foam cells in lipid metabolic dysregulation and immune response modulation. The increased presence of foam cells in the lung not only modulates macrophage polarization but also secretes pro-inflammatory cytokines (e.g., TNF-α, IL-1β) and fibrotic mediators (e.g., TGF-β1), thereby exacerbating local inflammation and promoting fibroblast migration and differentiation to drive fibrosis progression (Romero et al., 2015; Sun et al., 2024). Thus, foam cells play a complex role in pulmonary fibrosis development, contributing to disease progression through regulation of immune responses and lipid metabolism.

In summary, ferroptosis plays a pivotal role in modulating macrophage polarization, lipid metabolism, and foam cell formation in the fibrotic lung. Iron overload and lipid peroxidation drive functional reprogramming of macrophages, not only skewing their polarization toward M1 or M2 phenotypes but also impairing their lipid-clearance capacity to promote foam cell development. These changes amplify inflammatory responses and fibrotic remodeling, thereby establishing a critical pathological basis for the progression of pulmonary fibrosis.

### 7.4 Endothelial cell ferroptosis affects lung tissue regeneration and repair

Endothelial cells play a multifaceted role in the progression of pulmonary fibrosis, participating in disease onset and advancement through mechanisms such as barrier dysfunction, inflammatory cytokine release, and EndMT. Damage and death of endothelial cells lead to increased vascular permeability, thereby facilitating the infiltration of inflammatory cells and profibrotic mediators, which in turn activate fibroblasts and promote their differentiation into myofibroblasts (May et al., 2023; Zhao et al., 2023). On the other hand, endothelial injury exacerbates pulmonary inflammation and fibrotic remodeling, further advancing the disease. However, pulmonary endothelial cells also contribute positively to lung injury repair and regeneration. By secreting angiogenic factors, reconstructing the microvascular network, and maintaining oxygen supply and microenvironmental stability, they play a vital role in tissue restoration (Huang et al., 2023). Notably, emerging evidence indicates that healthy endothelial cells can not only participate in tissue repair but also suppress aberrant fibroblast activation, thereby attenuating fibrotic progression (Ackermann et al., 2024). These findings underscore the dual role of endothelial cells in the fibrotic microenvironment-while their damage and dysfunction drive disease progression, the preservation and restoration of their function exert protective and antifibrotic effects. Thus, maintaining endothelial homeostasis and modulating their functional balance has become a critical therapeutic strategy in pulmonary fibrosis. Approaches targeting the restoration of barrier integrity, promotion of endothelial repair and regeneration, and inhibition of EndMT may offer promising avenues for halting or reversing fibrotic progression.

Following lung injury, excessive accumulation of ROS and iron ions synergistically induces ferroptosis in pulmonary microvascular endothelial cells, leading to disruption of the vascular barrier. This process not only exacerbates local inflammation but also accelerates tissue fibrotic remodeling. Concurrently, elevated lipid peroxidation levels result in mitochondrial dysfunction within endothelial cells, characterized by mitochondrial shrinkage and disturbed energy metabolism. These changes further increase vascular permeability, promote infiltration of inflammatory cells, and worsen the progression of fibrosis (Sheng et al., 2021). In recent years, targeting endothelial ferroptosis has emerged as a promising therapeutic strategy for pulmonary fibrosis. Studies have shown that blocking the dopaminylation of triosephosphate isomerase 1 (TPI1) in regenerating endothelial cells can induce ferroptosis, activate perivascular fibroblasts, and inhibit alveolar epithelial regeneration. In contrast, restoring TPI1 dopaminylation effectively suppresses endothelial ferroptosis, promotes lung tissue repair, and significantly alleviates pulmonary fibrosis (Mo et al., 2024). These findings highlight the pivotal role of endothelial ferroptosis in the pathogenesis of pulmonary fibrosis and suggest that ferroptosis is a critical regulatory node linking lung injury repair and antifibrotic responses. Therefore, targeting iron homeostasis and ferroptotic pathways in endothelial cells may offer novel and effective strategies for the treatment of pulmonary fibrosis.

## 8 Ferroptosis-targeted strategies for pulmonary fibrosis

With the growing recognition of the pivotal role of ferroptosis in the pathogenesis of PF, therapeutic strategies targeting ferroptosis have become an emerging research focus. Numerous studies have demonstrated that modulating iron homeostasis, inhibiting lipid peroxidation, or enhancing cellular antioxidant defenses can effectively alleviate ferroptosis-associated cellular damage, thereby improving lung structure and function. In recent years, a growing number of studies have highlighted the promising potential of small-molecule compounds in regulating ferroptosis. These agents act through various mechanisms-such as restoring iron homeostasis, suppressing lipid peroxidation, or boosting antioxidant capacity-to reduce cellular injury and slow the progression of fibrosis. Several small molecules have already exhibited notable antifibrotic effects in animal models, offering new hope for clinical treatment of PF (Table 1). For instance, some compounds alleviate oxidative stress by modulating iron metabolism and inhibiting lipid peroxidation, thereby improving alveolar epithelial cell function and promoting lung tissue repair. Further preclinical and clinical studies are warranted to advance the development and application of these potential antifibrotic agents.

**TABLE 1:** Small-molecule drugs targeting ferroptosis regulation and their antifibrotic effects.

Therapeutic agents	Target	Preclinical data in PF
Deferoxamine	Iron chelator	Attenuated pulmonary epithelial cell death, mitochondrial damage, and pulmonary fibrosis (Cheng et al., 2021a; Takahashi et al., 2021) Reduced iron deposition and fibrosis levels in bleomycin-induced mouse lungs (Pei et al., 2022)
Ferrostatin-1	Antioxidation	Inhibits silica-induced ferroptosis and pulmonary fibrosis (Liu et al., 2022a; Liu et al., 2022c), and suppresses LPS-induced lung injury (Liu et al., 2020)
Liproxsrain-1	Antioxidation	Reduce collagen deposition and fibrosis markers in mouse lungs (Pei et al., 2022) Alleviate inflammation and restore redox balance (Li et al., 2019; Tao et al., 2021)
Deferasirox	Iron chelator	Reduced the cytotoxicity of *Pseudomonas aeruginosa* on airway cells (Moreau-Marquis et al., 2009)
Clioquinol	Iron chelator	Inhibited fibroblast activation, including proliferation, fibrotic differentiation, pro-inflammatory cytokine secretion, and migration (Zhu et al., 2021)
Rosiglitazone	ACSL4	Alleviates experimental pulmonary fibrosis by activating PPARγ and inhibiting TGF-β1 expression (Yu et al., 2017; Zhang et al., 2019a)
Pioglitazone	ACSL4	Inhibits BLM-induced acute lung injury and pulmonary fibrosis, reducing collagen production in the lung (Aoki et al., 2009; Grommes et al., 2012)
Troglitazone	ACSL4	Inhibits EMT of alveolar epithelial cells (Zhou et al., 2012). Inhibited fibroblast activation and collagen deposition (Milam et al., 2008)
Dihydroquercetin	ferritinophagy	Reduces intracellular lipid peroxides, inhibits ferritinophagy-induced ferroptosis, and alleviates SiO_2_-induced pulmonary fibrosis (Yuan et al., 2022). Upregulates SLC7A11 and GPx4 levels and inhibits lipid peroxidation in bronchial epithelial cells (Liu et al., 2022b)
Fraxetin	NCOA4	Inhibits the release of inflammatory cytokines in the lungs BLM-induced pulmonary fibrosis (Zhai et al., 2023)
Astragaloside IV	Nrf2/SLC7A11/GPX4	Restores GSH levels in lung tissue, reduces iron content in the lungs, and inhibits PM2.5-induced lung injury in mice (Wang et al., 2022b)

These studies offer a new perspective for the clinical treatment of PF and highlight targeting ferroptosis as an innovative therapeutic approach. With continued advances in the understanding of ferroptosis mechanisms and the development of small-molecule therapeutics, it is anticipated that more clinically applicable agents will emerge in the future. These novel therapies hold promise for expanding treatment options and improving the quality of life for patients with PF.

In addition to the aforementioned small-molecule compounds, several other agents with anti-ferroptotic properties have demonstrated significant anti-fibrotic effects in organs beyond the lung, suggesting their potential therapeutic value in pulmonary fibrosis. For instance, N-acetylcysteine (NAC), a well-known antioxidant, has been widely studied in various oxidative stress-related diseases (Santus et al., 2024). In experimental models of pulmonary fibrosis, NAC has been shown to alleviate disease progression by scavenging ROS, mitigating oxidative damage, and protecting alveolar epithelial cells, with its therapeutic potential validated by multiple studies (Mokra et al., 2023). Although direct evidence linking NAC to ferroptosis regulation in pulmonary fibrosis is currently lacking, NAC, as a precursor of GSH, can effectively replenish intracellular GSH stores, enhance GPX4 activity, and suppress lipid peroxidation. The anti-ferroptotic role of NAC has been well-documented in diseases of the liver and nervous system (Deepmala et al., 2015; Li et al., 2024b). Indicating that it may modulate ferroptosis-related processes in pulmonary fibrosis through similar mechanisms, thus offering a new direction for research into its application in this context. Moreover, several naturally derived compounds, such as sulforaphane, baicalin, and puerarin, have also been found to modulate ferroptosis indirectly through antioxidative activity, inhibition of lipid peroxidation, or maintenance of cellular iron homeostasis. These agents have been shown to reduce iron overload and oxidative stress, thereby attenuating tissue fibrosis progression in various disease models (Sun et al., 2016; Wang et al., 2021a; Huang et al., 2022; Wang et al., 2022a; Wen et al., 2023). The anti-fibrotic potential of these compounds suggests that combination strategies targeting ferroptosis may provide novel therapeutic avenues for the treatment of pulmonary fibrosis.

In summary, pharmacological strategies targeting ferroptosis-particularly the development of small-molecule inhibitors-offer a promising breakthrough for the treatment of refractory pulmonary fibrosis. However, the clinical application of these approaches still requires further validation. Critical aspects such as lung-specific drug delivery, bioavailability, and long-term safety remain to be thoroughly investigated and optimized.

## 9 Discussion and perspectives

Pulmonary fibrosis is a chronic interstitial lung disease characterized by progressive worsening and irreversible remodeling, driven by a highly complex interplay of multiple cell types and signaling pathways. In recent years, ferroptosis-a novel form of regulated cell death that is highly dependent on iron accumulation and lipid peroxidation has gained increasing attention in the study of pulmonary fibrosis. Emerging evidence indicates that ferroptosis exerts significant effects not only in alveolar epithelial cells, fibroblasts, macrophages, and vascular endothelial cells but also orchestrates a range of processes including oxidative stress regulation, immune response modulation, barrier dysfunction, and tissue remodeling. Notably, ferroptosis should not be viewed merely as a terminal cell death process, rather, it represents a dynamic and multifaceted pathological mechanism involving metabolic adaptation, signaling activation, and microenvironmental crosstalk, underscoring its central role in the onset and progression of pulmonary fibrosis.

At the cellular level, ferroptosis in alveolar epithelial cells impairs barrier function and gas exchange; in fibroblasts, it not only modulates cell activation and ECM production but may also create an “adaptive shield” that supports persistent fibroblast activation and a pro-fibrotic microenvironment. In macrophages, ferroptosis compromises their immune surveillance and inflammatory regulation capabilities, while in vascular endothelial cells, it can disrupt barrier integrity and exacerbate microenvironmental dysregulation. These intricate interactions across cell types and the matrix highlight the spatiotemporal heterogeneity of ferroptosis in different disease stages and cellular contexts. Although preclinical studies have demonstrated the potential of ferroptosis inhibitors and antioxidants to attenuate pulmonary fibrosis in animal models, translation to clinical applications faces significant challenges. First, the maintenance of iron homeostasis in lung tissue involves diverse cell types and dynamic metabolic reprogramming, leading to differential ferroptosis sensitivities across cell populations and disease stages-complicating precise therapeutic targeting. Second, ferroptosis rarely occurs in isolation; rather, it intersects with apoptosis, necrosis, and pyroptosis, with potential synergistic or antagonistic interactions. Therefore, future investigations must move beyond a ferroptosis-centric view to integrate it into a broader network of cell death and metabolic derangements to comprehensively elucidate its role in pulmonary fibrosis. Equally important is determining how to incorporate ferroptosis-targeted strategies into existing anti-fibrotic therapeutic frameworks such as those targeting TGF-β, oxidative stress, and inflammation to achieve synergistic, multi-target interventions that are more likely to yield clinical benefits.

Despite growing evidence implicating ferroptosis in the pathogenesis of IPF, and preclinical studies in cellular and animal models consistently link ferroptosis to fibrosis exacerbation, highlighting its significant contribution to alveolar injury and fibrotic remodeling in IPF. Nevertheless, current evidence does not support ferroptosis as a primary driver of the disease; rather, it should be regarded as a critical mechanism participating in the pathological process, rather than a singular central cause. Furthermore, the clinical and therapeutic applications of ferroptosis remain highly challenging. A major limitation is the lack of validated biomarkers to identify patients most likely to benefit from ferroptosis-targeted interventions or to monitor therapeutic efficacy. In addition, many ferroptosis inhibitors, including liproxstatin-1 and ferrostatin-1, exhibit poor pharmacokinetic properties, limited bioavailability, and suboptimal tissue targeting, complicating effective drug delivery to fibrotic lung regions. Safety concerns further constrain translation, as systemic ferroptosis inhibition may disrupt normal cellular homeostasis and produce off-target effects. Moreover, the complex interplay between ferroptosis and other cell death or fibrogenic pathways makes it difficult to predict therapeutic outcomes. Crucially, no large-scale, randomized clinical trials have yet demonstrated the efficacy or safety of ferroptosis-targeted therapies in IPF patients. Collectively, these limitations indicate that while ferroptosis represents a promising mechanistic target, substantial preclinical and translational work-including biomarker development, drug optimization, targeted delivery strategies, and rigorous clinical evaluation-is required before it can be effectively applied in clinical practice.

From our perspective, ferroptosis serves as a critical intersection between metabolic dysregulation and cell fate determination, providing a novel conceptual dimension for understanding disease pathogenesis. Ferroptosis should be considered not merely as a marker of cell injury but as a potential driver of disease progression itself. Accordingly, we advocate for expanding the research focus from single pathways to the dynamic “metabolism-oxidative stress-cell death” axis, identifying the coupled signaling hubs that may present precise therapeutic windows. Furthermore, advances in single-cell omics, spatial transcriptomics, and metabolomics technologies promise to generate high-resolution maps of ferroptosis within the heterogeneous pathological microenvironment of pulmonary fibrosis, paving the way for truly individualized and precise therapeutic strategies.

Finally, we emphasize that thorough preclinical pharmacodynamic and safety evaluations remain critical barriers to the clinical translation of ferroptosis-targeted therapies. Developing innovative molecules with higher tissue specificity, lower toxicity, and lung microenvironment compatibility will be essential for supporting precision medicine in pulmonary fibrosis. Intriguingly, exploring whether ferroptosis can act as an entry point to modulate metabolic adaptation and cell fate reprogramming offers a promising conceptual bridge from mechanistic understanding to precision intervention. We posit that systematically mapping the multidimensional interplay between ferroptosis and fibrosis progression, and integrating it with other pro-fibrotic signaling networks, will be crucial to advancing the next-generation of targeted pulmonary fibrosis treatments.
